# Supplementary material for: Global, quantitative and dynamic mapping of protein subcellular localization
Source: eLife. 2016 Jun 9;5:e16950. doi: 10.7554/eLife.16950 (PMC4959882; doi:10.7554/eLife.16950)
Supplement: Supplementary file 8. — An overview of the features and requirements of Dynamic Organellar Maps and the LOPIT approach. DOI: http://dx.doi.org/10.7554/eLife.16950.022 [file elife-16950-supp8.docx]

|  | **LOPIT**  Christoforou et al., 2016 | **Dynamic Organellar Maps**  Itzhak et al., 2016 |
| --- | --- | --- |
| Labeling strategy | Tandem Mass Tagging  (at peptide level) | SILAC metabolic labeling  (7 doublings in SILAC media) |
| Starting Material | 10^8^ Mouse ES cells | 10^7^ HeLa cells |
| Fractionation (time taken) | Density gradient centrifugation,  (18 hours 40 mins, excluding hands on time) plus detergent-based chromatin extraction | Differential centrifugation, (4 hours, including hands on time), including differential centrifugation for crude nuclear extract, reference membrane fraction and cytosolic fraction. |
| Multiplexing fractionation | unknown | Up to 4 maps in 4 hours with a single centrifuge |
| Preparation of peptides | In solution digestion, TMT labelling, C18 peptide cleanup, high pH reversed-phase liquid chromatography of combined labelled samples for 24 peptide fractions. | In solution digestion, SDB-RPS stage-tipping for cleanup, and eluting 1 or 3 peptide fractions per sample (8 samples, 5 SILAC, 3 label-free). |
| Mass spec run time | 24 x 105 min | 8 x 150 min for single peptide fraction, 24 x 150 min for 3 peptide fractions |
| Instrumentation | Obitrap Fusion, EASY-nLC 1000, required for MS^3^ | Q Exactive-HF, EASY-nLC 1000 |
| Organellar Resolution | Very good | Very good |
| Depth (proteins) | 5,500 (42 h measurement) | 6,000 (20 h measurement) 8,000 (60 h measurement) |
| Quantitative | No, only selected fractions analysed | Yes, all material analysed,  Copy numbers are extracted |
| Comparative applications | None published | Yes |

**Supplementary File 8** – **Comparison of organellar profiling approaches**
