# Supplementary material for: Global, quantitative and dynamic mapping of protein subcellular localization
Source: eLife. 2016 Jun 9;5:e16950. doi: 10.7554/eLife.16950 (PMC4959882; doi:10.7554/eLife.16950)
Supplement: Supplementary file 10. — DOI: http://dx.doi.org/10.7554/eLife.16950.024 [file elife-16950-supp10.pdf]

How to Use the Website www.MapOfTheCell.org

Username: guest
Password: cellmap2016

6. Click on the different maps to view where the protein was located in the individual replicates

2. Hover over points in the plot to identify them and click on them to reveal quantitative information.

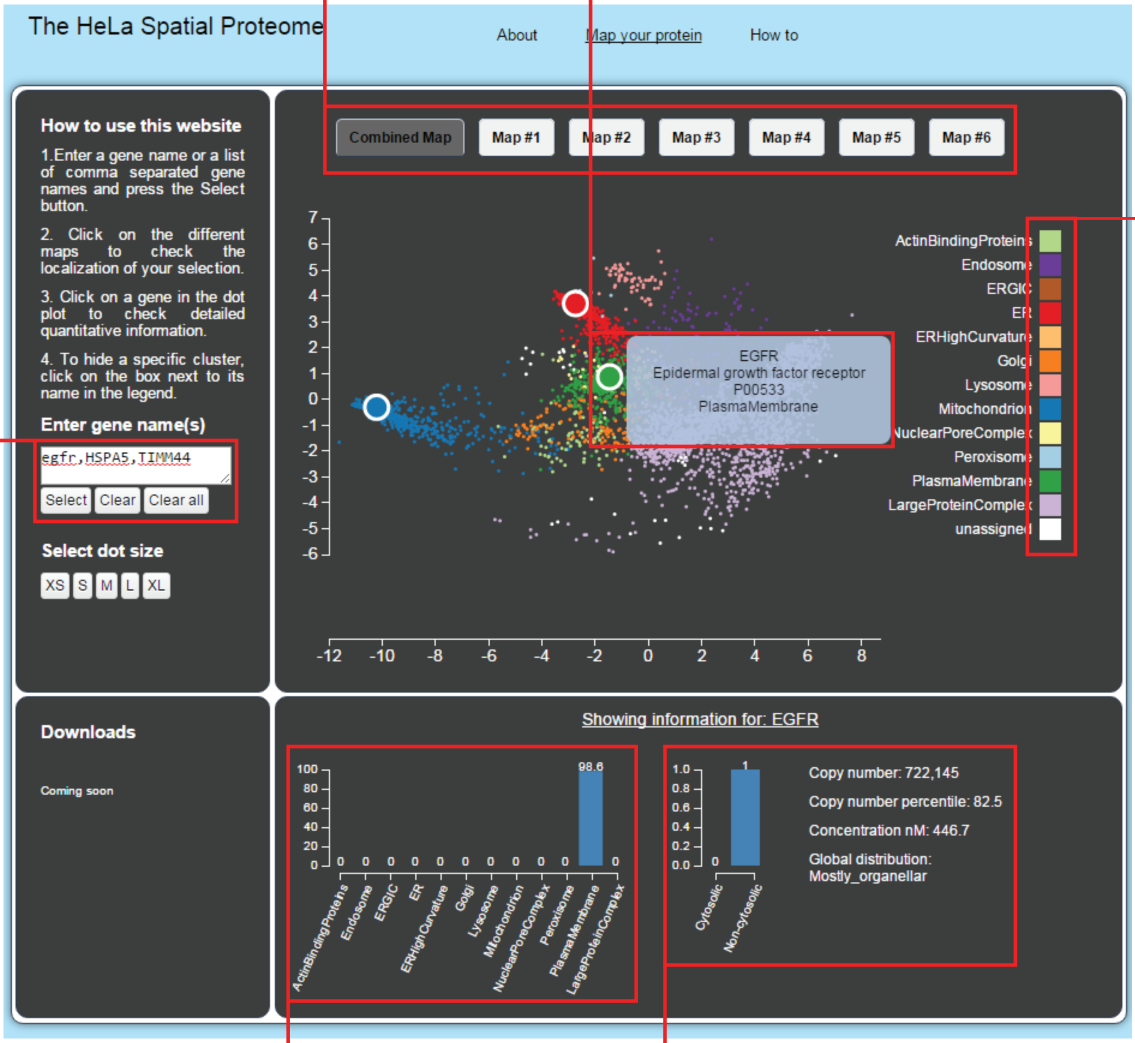

5. Click these buttons to hide specific clusters

1. Enter UniProt Gene names in this box separated by commas. Press select to highlight them on the plot

3. This plot reveals the organellar prediction score for the protein that was most recently clicked.

4. This shows the cytosolic pool of the protein, a verbal description of the global distribution as well as copy number
